# Activation and localization of matrix metalloproteinase-2 and -9 in the skeletal muscle of the muscular dystrophy dog (CXMD_J_)

**DOI:** 10.1186/1471-2474-8-54

**Published:** 2007-06-28

**Authors:** Kazuhiro Fukushima, Akinori Nakamura, Hideho Ueda, Katsutoshi Yuasa, Kunihiro Yoshida, Shin'ichi Takeda, Shu-ichi Ikeda

**Affiliations:** 1 Third Department of Medicine (Neurology and Rheumatology), Shinshu University School of Medicine, Matsumoto 390-8621, Japan; 2 Department of Molecular Therapy, National Institute of Neuroscience, National Center of Neurology and Psychiatry (NCNP), Kodaira 187-8502, Tokyo, Japan; 3 Department of Anatomy and Cell Biology, School of Health Sciences, Shinshu University, Matsumoto 390-8621, Japan

## Abstract

**Background:**

Matrix metalloproteinases (MMPs) are key regulatory molecules in the formation, remodeling and degradation of all extracellular matrix (ECM) components in both physiological and pathological processes in various tissues. The aim of this study was to examine the involvement of gelatinase MMP family members, MMP-2 and MMP-9, in dystrophin-deficient skeletal muscle. Towards this aim, we made use of the canine X-linked muscular dystrophy in Japan (CXMD_J_) model, a suitable animal model for Duchenne muscular dystrophy.

**Methods:**

We used surgically biopsied tibialis cranialis muscles of  normal male dogs (n = 3) and CXMD_J _dogs (n = 3) at 4, 5 and 6 months of age. Muscle sections were analyzed by conventional morphological methods and *in situ *zymography to identify the localization of MMP-2 and MMP-9. MMP-2 and MMP-9 activity was examined by gelatin zymography and the levels of the respective mRNAs in addition to those of regulatory molecules, including MT1-MMP, TIMP-1, TIMP-2, and RECK, were analyzed by semi-quantitative RT-PCR.

**Results:**

In CXMD_J _skeletal muscle, multiple foci of both degenerating and regenerating muscle fibers were associated with gelatinolytic MMP activity derived from MMP-2 and/or MMP-9. In CXMD_J _muscle, MMP-9 immunoreactivity localized to degenerated fibers with inflammatory cells. Weak and disconnected immunoreactivity of basal lamina components was seen in MMP-9-immunoreactive necrotic fibers of CXMD_J _muscle. Gelatinolytic MMP activity observed in the endomysium of groups of regenerating fibers in CXMD_J _did not co-localize with MMP-9 immunoreactivity, suggesting that it was due to the presence of MMP-2. We observed increased activities of pro MMP-2, MMP-2 and pro MMP-9, and levels of the mRNAs encoding MMP-2, MMP-9 and the regulatory molecules, MT1-MMP, TIMP-1, TIMP-2, and RECK in the skeletal muscle of CXMD_J _dogs compared to the levels observed in normal controls.

**Conclusion:**

MMP-2 and MMP-9 are likely involved in the pathology of dystrophin-deficient skeletal muscle. MMP-9 may be involved predominantly in the inflammatory process during muscle degeneration. In contrast, MMP-2, which was activated in the endomysium of groups of regenerating fibers, may be associated with ECM remodeling during muscle regeneration and fiber growth.

## Background

Duchenne muscular dystrophy (DMD) is the most common lethal X-linked recessive disease, presenting with progressive muscular atrophy and weakness. DMD is caused by mutations in the *DMD *gene that encodes the cytoskeletal protein dystrophin. Dystrophin and the dystrophin-associated protein complex provide a crucial structural link between the extracellular matrix (ECM) and the intracellular actin cytoskeleton [1]. Dystrophin deficiency affects the sarcolemma-ECM interaction, resulting in sarcolemmal instability [2,3]. Histopathological hallmarks in DMD include degeneration, necrosis, and insufficient regeneration of muscle fibers, suggesting that constitutive ECM remodeling takes place in DMD skeletal muscles. Although the cycles of degeneration and regeneration of muscle fibers continues throughout postnatal development, regeneration gradually slows and the balance is eventually tipped in favor of degeneration in DMD [4].

ECM is a three-dimensional network of macromolecules, and transmits signals from cells to the ECM and vice versa, mediating cell adhesion, migration, proliferation, differentiation and survival [5]. Muscle fibers are embedded in connective tissue organized into three interconnected sheaths: (1) Epimysium is a collagenous tissue that surrounds whole muscle; (2) Perimysium is smaller bundles of collagen fibers extended inward from epimysium, separates muscle fibers into fascicles or bundles; (3) Endomysium encloses the individual muscle fibers, including basal lamina, capillaries, fine nerve branches, fibroblasts, and macrophages [6]. The basal lamina, which consists of ECM components such as type IV collagen, laminin, and proteoglycans, maintains the physiological integrity of the muscle fibers and has a role in muscle fiber repair after injury or excessive exercise [7]. In the last decade, matrix metalloproteinases (MMPs) have been shown to degrade all ECM components [8]. MMPs, a group of zinc-dependent endopeptidases, are thought to play a central role in the modulation of ECM functions [9]. MMPs are commonly induced by cytokine signals as inactive zymogens (pro-forms), that require processing of a prodomain by other MMPs or serine proteinases to attain full activity. Their activities are inhibited by endogenous MMP inhibitors (tissue inhibitors of metalloproteinases; TIMPs-1, -2, -3, and -4) [10,11]. Membranous type metalloproteinases (MT-MMPs) have recently been discovered as a subgroup of membrane-anchored metalloproteinases. Membranous type metalloproteinase-1 (MT1-MMP) is associated with pro matrix metalloproteinase type 2 (pro MMP-2) and TIMP-2 to form a trimolecular complex, that activates pro MMP-2. [12-16]. Reversion-inducing-cysteine-rich protein with Kazal Motifs (RECK) is a new class of membrane-anchored inhibitor of MMP-2, matrix metalloproteinase type 9 (MMP-9), and MT1-MMP [17]. RECK has been described as a tumor and metastasis suppressor, as well as an angiogenesis suppressor and regulator of ECM integrity [18,19]. MMP activity contributes to a variety of physiological processes, such as embryonic development, organ morphogenesis, cell migration, apoptosis, angiogenesis, cartilage remodeling, bone growth, and wound healing [8,10,20]. On the other hand, loss of the exquisite regulation of MMPs leads to extensive ECM degradation, resulting in various diseases such as tumor progression or metastasis, cerebrovascular diseases, cardiovascular diseases, rheumatoid arthritis, and lung diseases. Therefore, MMP inhibitors can be useful prospective agents for the prevention and treatment of these diseases [21-27].

With respect to muscular disorders, particular attention has been paid to a subgroup of MMPs termed 'gelatinases,' comprising MMP-2 (also called gelatinase A, or 72-kDa type IV collagenase) and MMP-9 (also called gelatinase B, or 92-kDa type V collagenase). These enzymes contain three repeats of a gelatin-binding type II fibronectin domain inserted into their catalytic domain [28]. Besides gelatin, they degrade various ECM components including denatured type I, II, and III collagen, native IV and V collagen, laminin, elastin, proteoglycan, and fibronectin [28,29]. In some inflammatory myopathies, MMP-9 is expressed primarily by invading T lymphocytes, and is implicated in the pathogenesis [30-32]. MMP-2 is up-regulated in DMD skeletal muscle, and is expressed by mesenchymal fibroblastic cells [33]. Up-regulation of MMP-2 and MMP-9 has also been observed in skeletal muscle of dystrophin-deficient *mdx *mice [34,35], an animal model of DMD. Moreover, it has been reported that MMP-2 and MMP-9 are able to process beta-dystroglycan and disrupt the link between the ECM and the cell membrane via the dystroglycan complex in the skeletal muscle from DMD and sarcoglycanopathy patients [36-38].

We hypothesized that MMP-2 and/or MMP-9 might also play an important role in the pathogenesis of muscular dystrophies, involving ECM remodeling during the cycle of muscle fiber degeneration and regeneration. We evaluated the expression, activation, and immunolocalization of MMP-2 and MMP-9, as well as of regulatory molecules MT1-MMP, TIMP-1, TIMP-2 and RECK, in the dystrophin-deficient skeletal muscle of the canine X-linked muscular dystrophy in Japan (CXMD_J_) model of DMD, which shows more prominent skeletal muscle involvement than *mdx *mice [39].

## Methods

### Animals

CXMD_J _dogs were established by insemination of beagles with the sperm of golden retriever muscular dystrophy (*GRMD*) dogs [40]. CXMD_J _dogs lack dystrophin in the sarcolemma of skeletal muscles and exhibit typical dystrophic phenotypes, as observed in DMD and *GRMD *[39,41]. We studied three normal male dogs (Beagle) at 4, 5 and 6 months of age and three age-matched CXMD_J _dogs. Each of the CXMD_J _dogs presented with typical clinical signs of muscular dystrophy: progressive muscular atrophy and weakness, and elevated serum creatine kinase (CK) levels. The tibialis cranialis muscles of control and CXMD_J _dogs were obtained by surgical biopsy at the General Animal Research Facility of the National Institute of Neuroscience at the National Center of Neurology and Psychiatry (NCNP, Tokyo, Japan). The samples were rapidly frozen in isopentane cooled by liquid nitrogen. The treating and care of the dogs, and all experimental procedures, were approved by the Ethics Committee for the Treatment of Laboratory Middle-Sized Animal of the National Institute of Neuroscience, National Center of Neurology and Psychiatry (NCNP) (Tokyo, Japan). All experiments were performed with appropriate measures taken for preventing unnecessary pain.

### Film *in situ *zymography

To detect the localization of gelatinolytic MMP activity in skeletal muscle tissues, we performed film *in situ *zymography (FIZ) as previously described [42-48]. 6 μm-thick cryosections from frozen samples were placed on a polyethylene terephthalate base film coated with 7 μm thickness cross-linked gelatin with (FIZ-GI, Wako Pure Chemical, Osaka, Japan) or without (FIZ-GN, Wako Pure Chemical) 1,10-phenanthroline (inhibitor of MMPs, but not of other proteinases such as trypsin). The films with sections were incubated in a moist chamber for 6 hours at 37°C. Then the films were stained with Biebrich Scarlet for 4 min and rinsed for 10 min at room temperature. The degraded gelatin was not stained with Biebrich Scarlet, and areas of gelatinolytic activity were visualized as white or weakly red areas on the red background. Gelatinolysis observed on FIZ-GN films, but not on FIZ-GI films, indicated the presence of MMP activity. Gelatinolysis seen on both FIZ-GN and FIZ-GI films indicated the activity of proteases other than MMPs (such as trypsin). The application of gelatin as substrate for *in situ *zymography has the advantage that MMP-2 and/or MMP-9 have a high affinity for this substrate [49].

### Histopathology and immunohistochemistry

Serial 8 μm-thick cryosections of frozen muscle were prepared and stained with hematoxylin and eosin (HE). The sections were blocked with 10% goat serum (Cedarlane Laboratories, Burlington, Canada) in PBS for 15 min and incubated at 4°C overnight with primary antibodies, followed by secondary staining. Stained specimens were mounted in Vectashield with DAPI (4', 6-diamidino-2-phenylindole, dihydrochloride) (Vector Laboratories, Burlingame, CA) for nuclear counterstaining and photographed using a fluorescence microscope BX51 (OLYMPUS, Tokyo, Japan) equipped with an air-cooled CCD camera VB-7010 (KEYENCE, Oosaka, Japan) and VB-Viewer software (KEYENCE). Primary antibodies were used as follows: rabbit anti-MMP-2 antibody (AB809, Chemicon International, Temecula, CA), rabbit anti-MMP-9 antibody (AB19047, Chemicon International), mouse anti-CD11b antibody (MCA1777S, Serotec, Oxford, UK), mouse-anti myosin developmental type heavy chain (NCL-MHCd, Novocastra, Newcastle Upon Tyne, UK), mouse anti-laminin B2 antibody (MAB1920, Chemicon International), rat anti-heparan sulfate proteoglycan, perlecan antibody (MAB1948, Chemicon International), rabbit anti-TIMP-1 antibody (sc-5538, Santa Cruz Biotechnology, Santa Cruz, CA), rabbit anti-TIMP-2 antibody (sc-5539, Santa Cruz Biotechnology), goat anti-MT1-MMP antibody (sc-12367, Santa Cruz Biotechnology), and goat anti-RECK antibody (sc-8689, Santa Cruz Biotechnology). CD11b is a leukocyte-associated antigen expressed on monocytes, macrophages, and granulocytes [50]. Myosin developmental type heavy chain (MHCd) is a myosin heavy chain isoform, present during muscle fiber regeneration, in the embryonic and neonatal periods [51].

### Gelatin zymography

Frozen skeletal muscles were homogenized in an extraction buffer (62.5 mM Tris-HCl pH 6.8, 2% sodium dodecyl sulfate (SDS) 10% glycerol) and total protein content was assessed by the Bio-Rad DC Assay kit (Bio-Rad Laboratories, Hercules, CA). 100 μg of each extract dissolved in a loading buffer provided by the manufacture were electrophoresed through a gelatin-containing SDS polyacrylamide gel provided as part of the gelatin zymography kit (Gelatin Zymography kit, Yagai, Tokyo, Japan). The gel was washed with washing buffer and then incubated for 36 hours at 37°C in reaction buffer, provided by the manufacture. The gels were stained in Coomasie Brilliant Blue and destained with destain solution (Bio-Rad Laboratories). Gelatinolytic activity was identified as clear bands on a blue background. Gelatin zymography detects the activity of both active and pro-form gelatinolytic MMPs. This is because exposure to SDS during gel electrophoresis causes activation of the pro-form MMPs without proteolytic cleavage of the prodomain [52]. Quantitative results of the assays were obtained by densitometry using ImageJ v1.34s (National Institutes of Health; NIH, Bethesda, MD).

### RNA isolation and reverse transcription

Total RNA was isolated from the skeletal muscles using the RNeasy fibrous tissue mini kit (QIAGEN, Hilden, Germany) and RNA quality was spectrophotometrically assessed. Reverse transcription to cDNA was performed in buffered solution containing dATP, dCTP, dGTP, dTTP, FPLC*pure *murine reverse transcriptase, RNA guard (porcine), RNase/DNase-free bovine serum albumin, and *Not*I-d (T) 18 primer [5'-d(AACTGGAAGAATTCGCGGCCGCAGGAAT18)-3'] according to the manufacturer's instructions, using the Ready-To-Go T-Primed First Strand Reaction Kit (Pharmacia Biotech, Brussels, Belgium).

### Semi-quantitative RT-PCR

The PCR amplifications were carried out in the presence of 1.5 mM MgCl_2 _by using standard PCR buffer, 0.2 mM dNTPs, 0.4 mM of each forward and reverse primer, 1 μl of RT products, and 0.1 units Taq DNA polymerase in a total volume of 25 μl. We designed PCR primers based on the previously published cDNAs of the canine gene sequences for MMP-2, MMP-9, MT1-MMP, TIMP-1, TIMP-2, and RECK (Table 1). 18S ribosomal RNA was used as an internal control in the semi-quantitative RT-PCR experiments. A 324 bp fragment of 18s was amplified using QuantumRNA™ Classic II 18S Internal Standard (Ambion, Austin, TX). 18S rRNA primers were mixed with competimers at an optimized ratio of 5:5 so that the signal for 18S rRNA was attenuated to the level of messages of targets by modulating the efficiency of amplification of the 18S PCR product. The PCR conditions were: initial denaturation for 1 min at 94°C; followed by 30 cycles (for MT1-MMP, TIMP-1, and TIMP-2), or 32 cycles (for MMP-9 and RECK) of 94°C for 30 sec, 60°C for 30 sec and 72°C for 30 sec; and a final extension of 10 min at 72°C. The number of cycles was determined experimentally so that semi-quantitative comparison could be made during the exponential phase of the amplification process for each target and 18S rRNA gene. The relative expression level of each target gene was calculated by normalization against the 18s rRNA level. The PCR products were electrophoresed through a 2.5% agarose gel stained with ethidium bromide. The gel images were obtained using the GENE FLASH syngene bio imaging (TopoGEN, Port Orange, FL), and the densities of the products were quantified using ImageJ v1.34s (NIH). All assays were conducted in triplicate.

**Table 1:** Primer sets used for PCR

**Target gene **(GenBank accession No.)	**Primer set **(**Location of primer sequence)**
**MMP-2**	Forward 5'-TGCAAGGCAGTGGTCATAGCT-3' (1724–1744)
(XM 535300)	Reverse 5'-AGCCAGTCGGATTTGATGCT-3' (1829–1810)
**MMP-9**	Forward 5'-CCCTGCCACTTCCCCTTCACC-3' (707–727)
(AF 001003219)	Reverse 5'-GAGCGGCCCTCGAAGGTGAAC-3' (895–915)
**MT1-MMP**	Forward 5'-AGGAGACAAGCACTGGGTGTT-3' (38–58)
(AF 097638)	Reverse 5'-AGGGATTCCTTCCCAGACCTT-3' (249–269)
**TIMP-1**	Forward 5'-CCGACTTAAACCGGCGTTAT-3' (201–220)
(NM 001003182)	Reverse 5'-GATCAACACCTGCAGTTTCG-3' (401–420)
**TIMP-2**	Forward 5'-CAAAGCGGTCAGTGTGAAGG-3' (1–20)
(AF 095638)	Reverse 5'-CTTTGTGACTTCATCGTGCC-3' (236–255)
**RECK**	Forward 5'-TGCCCCGAAACAATGGTTGA-3' (246–265)
(NM 001002985)	Reverse 5'-TCTCGGCAGTTTGTGTGATGG-3' (494–514)

### Statistical analysis

Quantitative data were compared in both groups by Mann-Whitney's U test. The data are presented as mean ± SEM. *P *< 0.05 was considered to indicate statistical significance.

## Results

### Histopathology and *in situ *zymography

**Figure 1:**
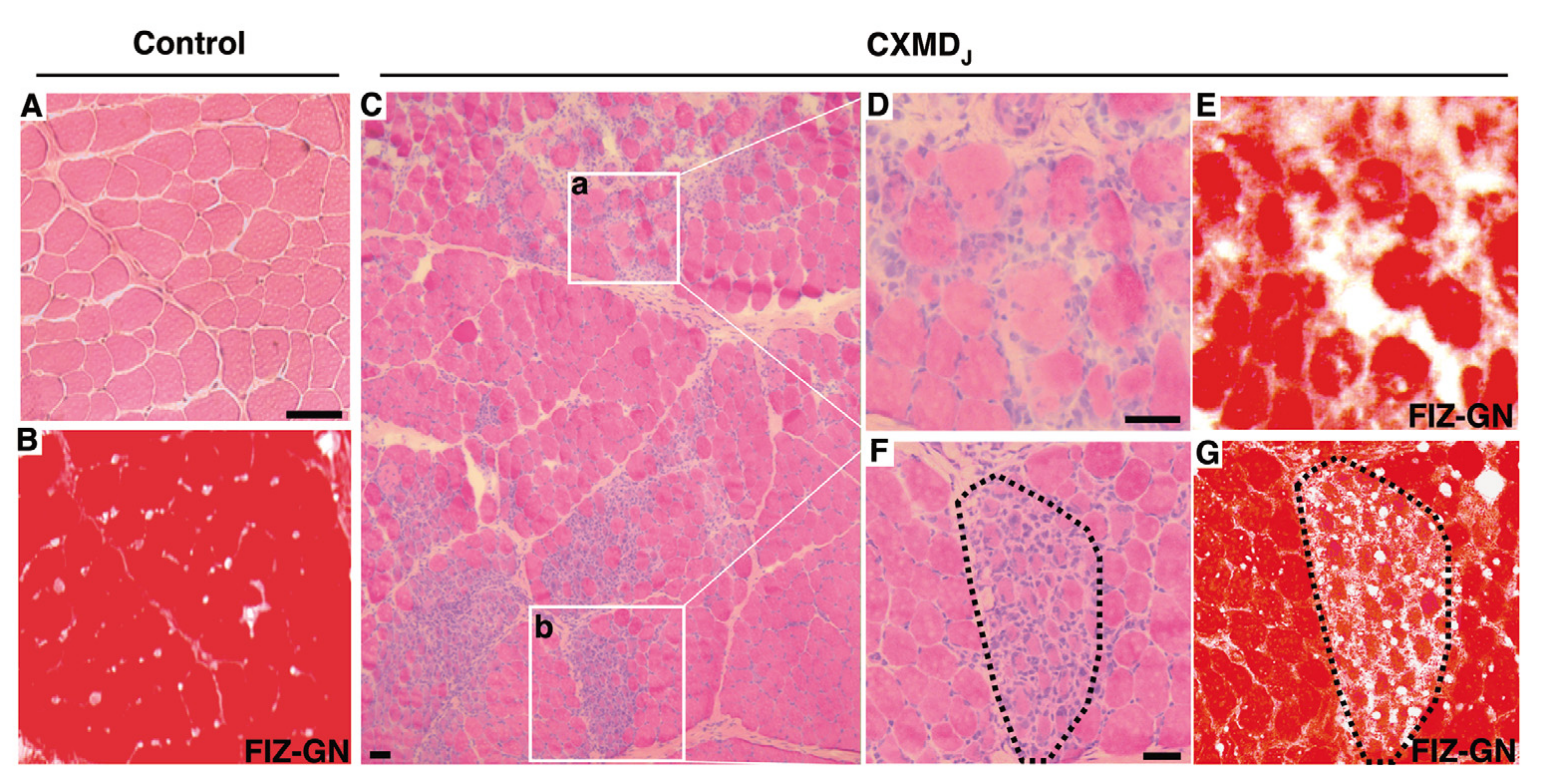
**Film *in situ *zymography (FIZ-GN) of skeletal muscle in CXMD_J _dogs**. Hematoxylin and eosin (H&E) staining (A, C, D, and F) and film *in situ *zymography (FIZ-GN) (B, E, and G) of the tibialis cranialis muscle in a normal dog (A, B) and a CXMD_J _dog (C-G). D and E (magnified views of C-a): a representative area of massive necrotic fibers with inflammatory infiltrate. F and G (magnified views of C-b): a representative area of a group of regenerating fibers. Gelatinolytic activities were visualized as white or weakly red areas on the red background. Bars, 100 μm.

CXMD_J _muscles showed typical histopathological hallmarks of degeneration and regeneration. Multiple foci of both degenerating and regenerating muscle fibers were observed simultaneously in the same section (Fig. 1C). We examined the localization of gelatinolytic activity in the skeletal muscle of control and CXMD_J _dogs. In controls, low gelatinolytic activity was detected in the endomysium (Fig. 1A,B). In contrast, strong activity was detected in the endomysial space with infiltrating inflammatory cells in CXMD_J _muscles (Fig. 1D,E). Moreover, the endomysium of groups of regenerating fibers showed prominent gelatinolytic activity in CXMD_J _muscles (Fig. 1F,G).

**Figure 2:**
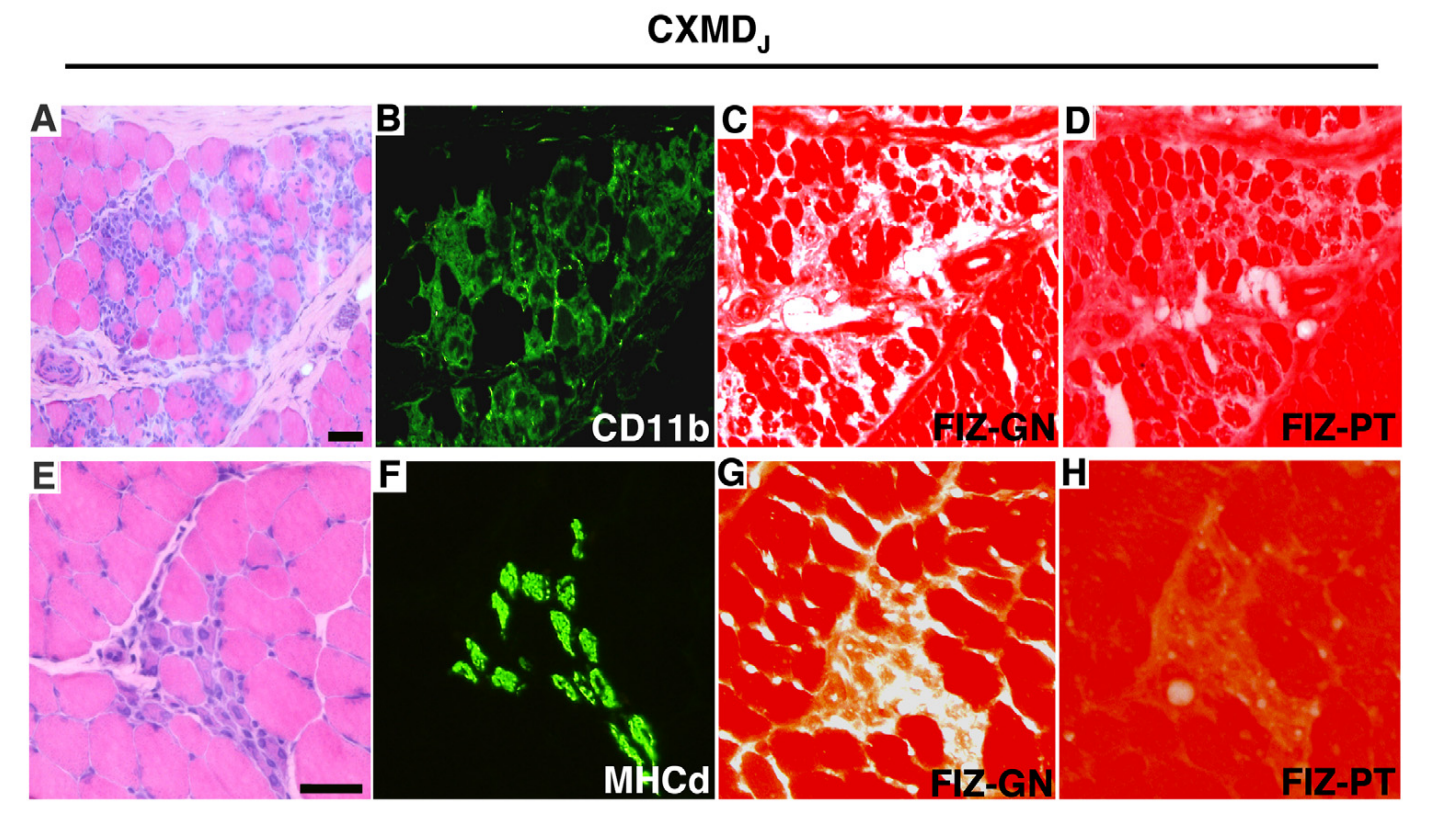
**Immunohistochemical analysis of CD11b or MHCd expression, and film *in situ *zymography in the presence and absence of an MMP inhibitor (FIZ-PT) in the skeletal muscle of CXMD_J _dogs**. A-D: serial sections of CXMD_J _muscle characterized by extensive necrosis and an invading inflammatory infiltrate; E-H: serial sections of groups of regenerating fibers in CXMD_J _muscle. A and B: H&E staining; B and F: immunohistochemical analysis of CD11b; C and G: FIZ-GN; D and H: FIZ in the presence of an MMP inhibitor (1,10-phenanthrolone) (FIZ-PT). Gelatinolytic activity co-localized with CD11b and MHCd, immunoreactivity and was attenuated by adding 1,10-phenanthrolone to the FIZ assay system. Bars, 50 μm.

We next examined the fibers characterized by prominent gelatinolytic activity using immunohistochemistry. Strong gelatinase activity was detected in the endomysium not only of fibers with infiltration of CD11b positive cells (Fig. 2A,B,C), but also of MHCd-positive, small caliber, centronuclear fibers (Fig. 2E,F,G), which represent degenerating and regenerating fibers, respectively. Gelatinolytic activity was attenuated by the MMP inhibitor, 1,10-phenanthrolone (Fig. 2D,H), indicating that it was specifically derived from MMPs, and not from other proteases.

### Immunohistochemistry of MMP-9

**Figure 3:**
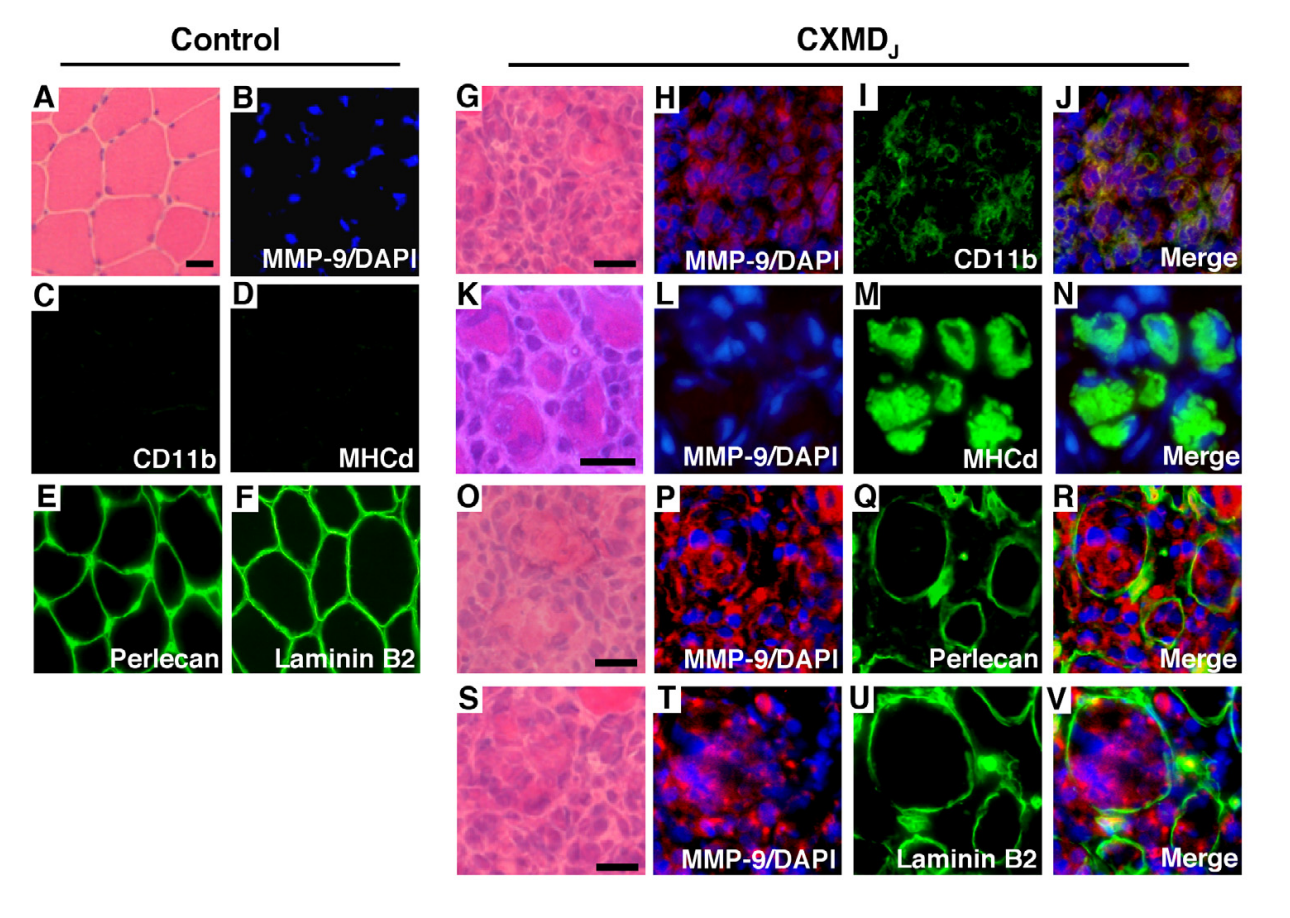
**Co-immunolocalization of MMP-9, Perlecan, and laminin B2 in the skeletal muscle of CXMD_J _dogs**. H&E staining (A, G, K, O and S). Immunohistochemical analysis of MMP-9 (red), with nuclear counterstain using DAPI (blue) (B, H, L, P and T), CD11b (green, C and I), MHCd (green, D and M), Perlecan (green, E and Q), laminin B2 (green, F and U). Merged images of MMP-9 immunoreactivity with that of CD11b (J), MHCd (N), Perlecan (R) and laminin B2 (V) in normal control (A-F) and in a CXMD_J _muscle (G-V). MMP-9 was detected in necrotic fibers associated with invading inflammatory cells and co-localized with CD11b (G-J). The endomysium of surrounding MHCd-positive regenerating fibers showed no MMP-9 immunoreactivity (K-M). A fraction of the MMP-9-positive fibers had degenerated and showed a weak and discontinuous pattern of immunostaining of both perlecan (O-R) and laminin B2 (S-V). Bars, 25 μm.

In CXMD_J _muscle, MMP-9 immunoreactivity was detected in the necrotic fibers with inflammatory cell invasion and their endomysial space. MMP-9 immunoreactivity was co-localized with that of CD11b (Fig. 3G–J). Some MMP-9-positive fibers had degenerated and showed a weak and discontinuous immunoreactivity pattern of basal lamina components, perlecan (Fig. 3O–R) and laminin B2 (Fig. 3S–V). On the other hand, regenerating fibers (MHCd-positive fibers) in CXMD_J _muscle did not show MMP-9 immunoreactivity (Fig. 3K–N). Control muscle did not show a significant immunoreactivity for MMP-9, CD11b and MHCd (Fig. 3B,C and 3D, respectively), and revealed a normal immunoreactivity of perlecan and laminin B2 (Fig. 3E and 3F, respectively). Each immunohistochemistry of MMP-2, MT1-MMP, TIMP-1, TIMP-2, or RECK failed because of poor immunoreactivity of each antibody to the canine isoforms.

### Gelatin zymography

**Figure 4:**
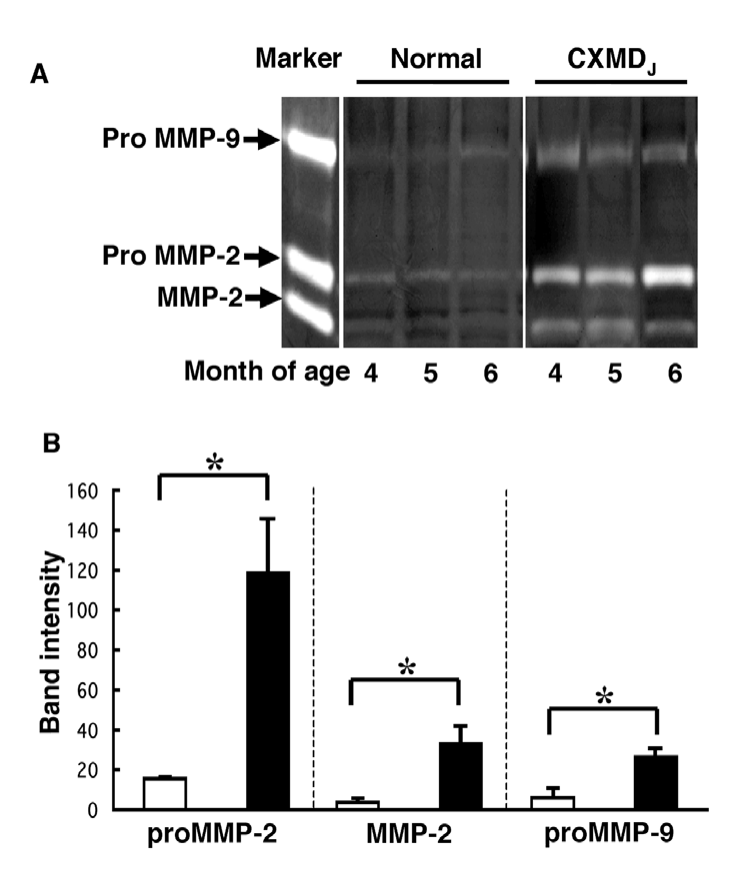
**Gelatinolytic activities assessed by zymography in the muscle of normal and CXMD_J _dogs**. A. Equal amounts of protein (100 μg) extracted from tibialis cranialis muscle was loaded onto a gelatin-containing SDS polyacrylamide gel. Gelatinolytic activity was identified as clear bands on a background. The activities of MMP-2, pro MMP-2 and pro MMP-9 were increased in the muscles of CXMD_J _dogs (n = 3) compared with those in the muscle of normal control dogs (n = 3). B. Semi-quantitative analysis of the activities of pro MMP-2, MMP-2 and pro MMP-9 in control (open bar, n = 3) and CXMD_J _(closed bar, n = 3) dogs based on analysis of the band intensity associated with each activity. Bar: mean ± SEM; *p < 0.01.

We next evaluated the activity of each of the gelatinolytic enzymes (MMP-2, pro MMP-2 and pro MMP-9) in the muscle of normal and CXMD_J _dogs by gelatin zymography analysis. This analysis detected gelatinolytic enzyme activity associated with proteins of molecular weights corresponding to 74 kDa, 64 kDa and 92 kDa, which indicate the presence of pro MMP-2, and the active forms of MMP-2 and pro MMP-9, respectively. The activities of the three enzymes, particularly pro MMP-9, were increased in CXMD_J _muscle compared to control samples. MMP-9 was only detected as the pro-form (Fig. 4A). A semi-quantitative analysis showed a significant difference in each MMP activity between CXMD_J _and normal dog muscle (Fig. 4B).

### Semi-quantitative RT-PCR

**Figure 5:**
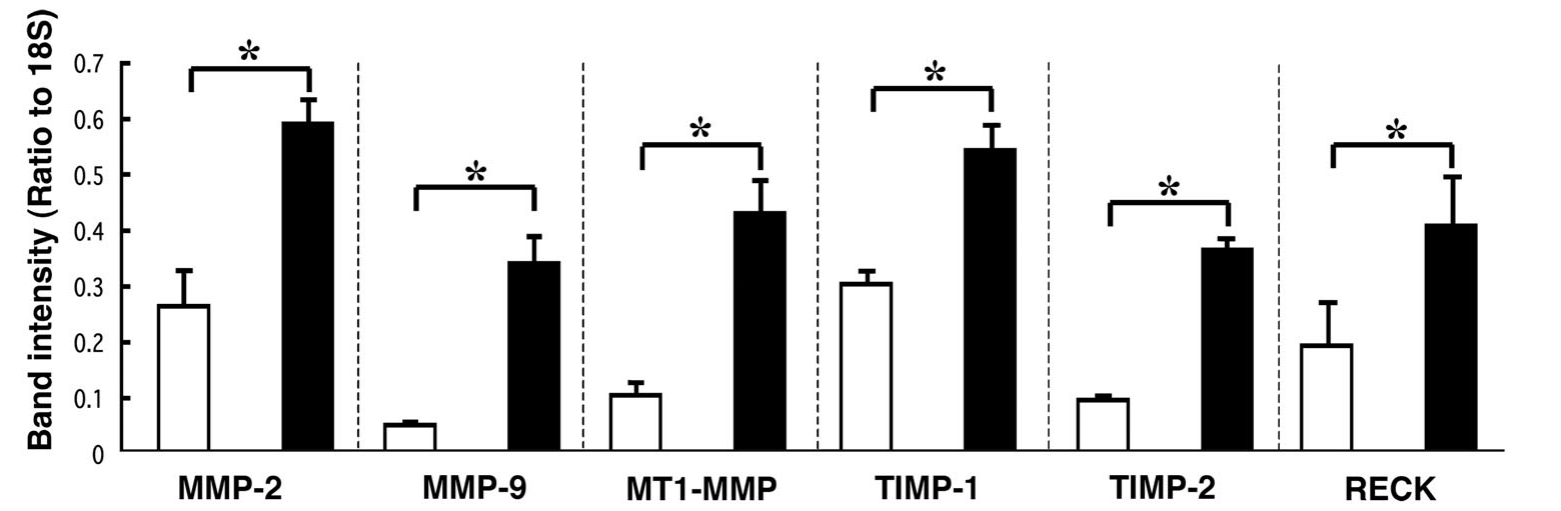
**Relative mRNA levels of MMPs, endogenous MMPs inhibitors, and RECK in muscle from normal and CXMD_J _dogs**. The mRNA levels of MMP-2, MMP-9, MT1-MMP, TIMP-1, TIMP-2, and RECK were examined by semi-quantitative RT-PCR. The control dogs (open bar, n = 3), CXMD_J _(closed bar, n = 3). Bar: mean ± SEM; *P < 0.01.

We next analyzed each gene expression of the gelatinolytic enzymes or the associated regulatory molecules in control and CXMD_J _dog muscle. A semi-quantitative analysis of the mRNA level encoding MMP-2, MMP-9, MT1-MMP, TIMP-1, TIMP-2, and RECK revealed that they were significantly increased in CXMD_J _muscle compared to the levels in control muscle (Fig. 5).

## Discussion

Our data suggest that ECM remodeling mediated by MMP-2 and MMP-9 is a key process in skeletal muscle fiber degeneration and regeneration in CXMD_J _dogs. An FIZ assay revealed multiple foci of both degenerating and regenerating muscle fibers associated with gelatinolytic activity that was attenuated by exposure of the tissue to an MMP inhibitor, 1,10-phenanthrolone, indicating the presence of the activity of the gelatinolytic subgroup MMPs, MMP-2 and MMP-9. We also observed increased MMP-2 and MMP-9 activity and expression using gelatin zymography and by measuring the corresponding mRNAs, respectively. In addition, we observed increased expression of the regulatory molecules, TIMP-1, TIMP-2, RECK, and MT1-MMP. Overall proteolytic activity is not easily predicted by RT-PCR findings on MMPs and their regulators, but we performed FIZ to know total gelatinase activities *in situ*. Our results showed that two proteases, MMP-2 and/or MMP-9 were highly activated in certain areas, although the data was not quantitative.

We showed that MMP-9 was localized to the endomysial space of inflammation in CXMD_J _muscle. MMP-9 seems to be derived from infiltrating granulocytes, monocytes or macrophages, since MMP-9 and CD11b immunoreactivity appear to co-localize. Degenerating fibers associated with MMP-9 immunoreactivity exhibited weak and disconnected expression of basal lamina components, laminin B2 and perlecan. These results suggest that MMP-9 may promote the degradation of the basal lamina in necrotic fibers in CXMD_J _muscle, followed by inflammatory cell invasion. In cardiotoxin-injected mice, MMP-9 was induced within 24 h of injection into skeletal muscle and was associated with inflammatory cell invasion, and MMP-9 mRNA was localized to invading polymorphonuclear cells and macrophages [34]. In human inflammatory myopathies, it has been reported that MMP-9 is produced primarily by invading T lymphocytes [30-32]. It is conceivable that MMP-9 may also contribute to elimination of necrotic fibers, so as to make room for muscle regeneration. MMP-9 is reported to be associated with muscle satellite cells; an adult muscle precursor and candidate muscle stem cell, and cultured human muscle satellite cells treated with phorbol ester [53]. MMP-9 mRNA was expressed in putative activated satellite cells in injured mouse muscle [34]. Taken together, these data suggest that MMP-9 might be associated not only with ECM degradation during inflammation, but also during the initiation of muscle regeneration. In our experiments, we detected only the latent form of MMP-9 (pro MMP-9) in the gelatin zymography. Regarding MMP-9, it has been reported that MMP-9 is not always detected in many tissues in vivo, although pro MMP-9 is converted to MMP-9 by various protease such as MMP-1, MMP-2, MMP-3 or plasminogen activator in vitro [54]. In addition, MMP-9 activation locally occurs in a pericellular environment by inflammatory response, leaving much of the secreted MMP in its pro-form [55].

Our data indicated that MMP-2 might be activated in the endomysium of regenerating fibers in CXMD_J _muscle. The endomysium of regenerating fibers were associated with gelatinolytic MMP activity, but were not associated with MMP-9 immunoreactivity. These results suggest that MMP-2, but not MMP-9, is activated in the vicinity of regenerating fibers. In a previous report, MMP-2 transcripts were predominantly found in the areas of fiber degeneration and ECM regeneration, and were localized to mesenchymal fibroblasts in DMD skeletal muscle [33]. During muscle degeneration and regeneration induced by cardiotoxin injection, MMP-2 activity seems to be increased concomitantly with the transition from the regeneration phases characterized by the appearance of young myotubes to maturation of the myotubes into multinucleated myofibers [34,56]. We therefore, assume that MMP-2 may take part in ECM remodeling occurring in the endomysium of regenerating fibers, as they grow into mature fibers. Additionally, there are some reports indicating that MMP-2 is involved in earlier phases of muscle regeneration. In the mouse myoblastic C2C12 cell line, MMP-2 and MT1-MMP promote myoblast fusion [57], and MMP-2 could degrade the basal lamina components during myoblast fusion [40].

TIMPs, endogenous tissue inhibitors of MMPs, have been considered to be involved in the activities of MMPs. The expression of TIMPs has been explored in some diseased skeletal muscle, but their results were not conclusive. One report mentioned that expression of TIMP-1 and TIMP-2 mRNA levels were not different between inflammatory myopathies/inclusion body myositis and muscular dystrophy [32]. While, it has been also reported that both TIMP-1 and TIMP-2 mRNA levels in DMD muscle were increased than those in normal or other pathological controls, resulting in the promotion of muscle fibrosis [33]. Therefore, it is valuable to compare expression of TIMPs in dystrophin-deficiency and those in experimental models for muscle degeneration-regeneration. In our study, we presented up-regulation of TIMPs in the same skeletal muscle from CXMD_J _at 4 to 6 months of age, where both necrosis and regeneration was active and existed at the same time. In injured mouse skeletal muscle by cardiotoxin injection, the increase of TIMP-1 mRNA levels was noticed a little later than the peak of MMP-9 mRNA, whereas TIMP-2 mRNA levels were found corresponding to the increase in MMP-2 mRNA (Fukushima *et al*., unpublished observations). We, therefore, suppose that MMPs and TIMPs upregulation may depend on the stage of muscle pathology; muscle necrosis or muscle regeneration, although cardiotoxin injection into canine skeletal muscle would allow us to directly compare muscle regeneration process and pathology of muscular dystrophy.

## Conclusion

Based on our study, MMP-9 may be predominantly involved in the inflammatory process during muscle degeneration, while MMP-2 may be associated with ECM remodeling during muscle regeneration and fiber growth. To further elucidate the specific functions of each MMP and TIMP, canine muscle injury model by injection of a mytotoxic agent would be largely informative and therefore indispensable.

## Competing interests

The author(s) declare that they have no competing interests.

## Authors' contributions

KF carried out the molecular and pathological examination, and drafted the manuscript. AN participated in the design of the study and drafted the manuscript. HU performed pathological analyses. KY participated in the maintenance of the dogs and necropsy. KY participated in the molecular analyses. ST participated in the design and coordination of the study. SI participated in the planning and coordination of the study. All authors read and approved the final manuscript.

## Pre-publication history

The pre-publication history for this paper can be accessed here:


